# Potential and challenges of high-speed (4D) body scanning for mobility analysis of firefighter clothing: a methodical case study

**DOI:** 10.17179/excli2023-6101

**Published:** 2023-10-19

**Authors:** Dominik Muenks, Yordan Kyosev, Felix Kunzelmann

**Affiliations:** 1 Chair of Development and Assembly of Textile Products, ITM, TU Dresden, Germany

**Keywords:** highspeed body scanning, motion comfort, automated analysis, protective clothing, firefighter clothing, 4D-scanning

## Abstract

In this study, protective clothing for firefighters is analyzed using 4D body scanning and 3D hand scanning, with a focus on the experimental analysis of ergonomic comfort. In particular, German firefighting clothing is examined to discuss the possibilities and limitations of current scanning technologies for capturing firefighting clothing. For this purpose, various movements are recorded in the 4D scanner. In addition, a method for determining position changes of protective clothing at identified limits is presented. The initial results illustrated that the analysis of protective clothing for firefighters using 4D scanning is problematic due to specific materials, reflections, and surface properties. Improvements in the scanning process and optimization of algorithms are required to achieve more detailed and precise results. Concerning the ergonomic comfort related to the mobility under firefighting clothing use conditions, this methodical case study highlights the limits of current approaches, with a focus on the limitations of 4D scanning and potential improvements.

## Introduction

Comfort in clothing refers to the sense of well-being and satisfaction that a wearer feels when wearing a garment and is subdivided into various factors for assessing comfort, including thermophysiological, skin-sensory, ergonomic and psychological comfort (Hohenstein Institut für Textilinnovation gGmbH, 2023[13]). Whereby the human body and clothing interact in different ways. The thermal exchange is important for the human body comfort (Salopek Cubric et al., 2021[28]) and depends on the materials and on the clothing construction. The gap between the body and the clothing is investigated by 3D body scanning for single postures (Lu et al. 2014[17]), and changes significantly during the motion (Xiaohui et al., 2011[31]), which might influence the freedom of movement under protective clothing use conditions (EN 17558, 2023[7]).

In recent years, there have been many improvements in the field of protective clothing in terms of the materials used and the fit. The opposing requirements for improving the thermal protection of and reducing the heat stress on the wearer of firefighting protective clothing will require balancing (Havenith, et al., 1999[12]). Overall, however, comfort is a secondary concern in firefighting clothing compared to the protective function of the clothing. The focus is on ensuring that the equipment is designed to provide firefighters with the necessary safety and protection to be able to work effectively in hazardous operational situations. An important component of firefighter clothing is heat-resistant gear, composed of several layers of fire-retardant materials such as NOMEX® Tough, NOMEX®, NXT, NOMEX® XTR, PBI and highly heat- and flame-resistant materials (PU-coated aramid fabric) (Rosenbauer International AG, 2023[27]).

The two studies by Ciesielska-Wróbel et al. (2017[2], 2018[3]) conclude that the design of protective uniforms has a significant influence on the performance of firefighters during moderate physical exercises. The authors emphasize the importance of careful design of protective uniforms to ensure firefighter performance and safety. They note that both the fit of the uniforms and the materials used play an important role in improving the comfort and freedom of movement of firefighters during their missions (Ciesielska-Wróbel et al., 2017[2]). The authors suggest that improving the fit and design of operational uniforms can help to improve firefighters' performance and reduce the risk of injury and fatigue (Ciesielska-Wróbel et al., 2018[3]).

A good clothing fit not only protects the wearer, but also ensures that overheating does not occur. The microclimate within the clothing is important for the wearer's thermophysiological comfort, as it maintains an optimal core body temperature and protects the body from overheating or undercooling. In garments, the microclimate refers to the climatic conditions close to the wearer's body, which are influenced by the garment. The microclimate in clothing can be influenced by various factors, such as the breathability of the clothing, thermal insulation, air permeability, and moisture regulation. Poor air permeability or insufficient moisture regulation in clothing can negatively affect the microclimate within the clothing, leading to discomfort or even health problems (Thorns, 2009[29]). Psikuta et al. (2023[26]) showed that differences in the distribution of air gaps (distance between clothes and the body) in firefighters' clothing occur according to body pose. Significant changes in air layer thickness were found on the upper and lower body depending on body posture, which has an effect on thermal as well as evaporative resistance and consequently also on potential thermal performance (Psikuta et al., 2023[26]). Even small improvements, such as optimizing air gap properties can improve the thermal protection of firefighting clothing and reduce the risk of burns and heat stress for firefighters (Deng et al., 2018[5]). 

Grabowska et al. (2020[8][9]) investigated the possibility of customizing protective clothing based on 3D scanning technology. The results showed that the customization of protective clothing based on 3D scanning leads to improved fit and function of the garments. The study concluded that using 3D scanning techniques to customize protective clothing is a promising approach that can lead to better fit, increased comfort and better performance for the user (Grabowska et al., 2020[8][9]). In addition, many other studies are looking at using 3D scans to investigate the fit between men and women. The aim is to adapt and improve protective clothing accordingly (McQuerry, 2020[18]; Park and Langseth-Schmidt, 2016[25]). Using 3D scanning the protection area of the clothing can be evaluated (Muenks et al., 2022[22]). All these methods and foundations would provide more useful information for the mobility if they were applied for a series of scans, so that the complete motion sequences can be analyzed and the changes of the body form as well as required clothing dimensions can be obtained (Kyosev et al., 2022[14]). Searching the literature, we could not identify any studies which used 4D scans to examine personal protective equipment.

### Aim

Protective clothing is usually made of special materials where the protective function comes first and comfort is secondary. To ensure adequate protection, the textiles are usually coated (for chemical protection), combined in several layers (e.g., for bullet or abrasion protection) or fitted with rigid elements, e.g., for stab protection (Muenks et al., 2022[23]). All these additional layers or elements increase the stiffness of the material and significantly restrict the freedom of movement. This paper presents a method for the experimental analysis of the movement comfort of protective clothing using 4D body scanning at high speed. In particular, the German fire brigade protective clothing is considered. The possibilities of measuring firefighters' clothing using 3D and 4D body scanning are investigated. The aim is to establish a method to determine the ergonomic comfort of the user of firefighters' clothing and to show the limitations of the current methodology. This study focuses on initial measurements showing the limitations of 4D scanning and developing possible improvements.

## Materials and Methods

### Firefighter protective clothing

German fire fighter clothing (NOVOTEX-ISOMAT Schutzbekleidung GmbH) was used. The protective clothing of type NTi®-112 Model 1 Premium consists of a jacket and trousers with braces. The firefighter clothing is certified to comply with the standard EN 469:2005+A1:2006+AC:2006 and the performance levels Xf2/Xr2/Y2/Z2. The EN 469:2005 standard was replaced by EN 469:2020 (Hanseatische Feuerwehr-Unfallkasse Nord, 2021[11]). After consultation with the manufacturer, there have been no changes to the design and materials, so the clothing complies with current regulations. The outer fabric is made of Nomex® Outershell Tough (75 % meta-aramid (Nomex®)/23 % para-aramid (Kevlar®)/2 % antistatic fibre) and the insulation layer/lining is made of Nomex®/ Kevlar® quilted with 50 % aramid/50 % viscose FR (NOVOTEX-ISOMAT Schutzbekleidung GmbH, 2023[24]). A helmet was also included with the manufacturer unknown. According to personal communication with the fire brigade, it is common practice to wear normal street clothes under the fire brigade clothing. According to Wang et al. (2013[30]), the type of clothing worn under the firefighters' clothing is crucial and has a significant influence on thermal and moisture comfort. Wang's studies conclude that cotton or linen innerwear has better thermal and moisture comfort than polyester innerwear (Wang et al., 2013[30]). Therefore, in this study, a 100 % cotton (BW) T-shirt was worn underneath the jacket and jeans with a composition of 98 % BW and 2 % elastane (EL) were worn underneath the trousers.

Initial measurements were performed with a male participant aged 40 years. Unfortunately, only one size of protective clothing was available for the studies. The clothing size of the jacket is 50/52 with a recommended body height of 174-178 cm and a chest circumference of 126-132 cm. The size of the trousers is L (50/52) with a recommended height of 178-182 cm and a waist of 102-106 cm. It should be noted that the protective clothing did not fit the participant perfectly (body height 183 cm, chest circumference 105 cm, waist 98 cm). This suggested that the jacket was too short for the participant, but too large in the chest area. The trousers were about 1 cm too short but too large in the waist area. This can have affected the mobility of the wearer. Although this was not considered decisive for the general presentation of the methodology, future applications should use appropriately fitting clothing when mobility will be examined.

### Scan methods

The tests were performed using the 4D scanning system of MOVE4D, which was developed by the Instituto de Biomecánica de Valencia (IBV) and was installed in the scanning laboratory of the TU Dresden. The 4D scanner can produce up to 178 3D scans per second with a distance resolution of 1-2 mm (Move 4D, 2023[21]). For normal movement, the scan frequency of 30-45 scans provides sufficient detail to analyze the movement of the body in interaction with the clothing. For clothes for martial arts (karate, etc.), 60-90 images per second are required. After scanning, one obtains a series of files with point clouds that can be analyzed with different methods. In the current study, the point cloud distances were used to analyze the relative movement of the clothes. Nearest neighbor distance metrics (Clark & Evans, 1954[4]) was intensively employed as methods for comparing point clouds over the last 20 years (Mémoli & Sapiro, 2004[20]). One of the most commonly used distances is the Gromov-Hausdorff distance (Memoli, 2007[19]), which is implemented in several cloud analysis packages such as Meshlab Filter (Paolo Cignoni, Visual Computing Lab, ISTI-CNR), CloudCompare (CloudCompare) or in Matlab (The MathWorks, Inc.).

In the course of the investigation, it is found that the 4D scanner reaches its limits in certain situations and materials in connection with firefighters' clothing, which are explained in more detail in the “Results” chapter. For this reason, an Artec Leo 3D hand-held scanner is also used. The handheld scanner has a 3D point accuracy of 0.1 mm, with a 3D resolution of 0.2 mm and a 3D accuracy over a distance of up to 0.1 mm + 0.3 mm/m (Professional 3D scanning solutions | Artec 3D, 2023[1]).

**Figure 1:**
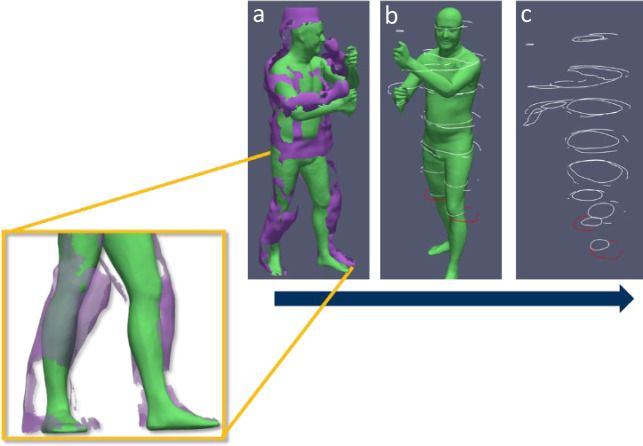
Joined frames in a) mesh to mesh; b) mesh to cross-section and c) cross-section to cross-section view

To examine the resulting air gaps, a test person is 4D scanned at different levels. The test person is first scanned in underwear close (first level) to the body and then in full firefighting gear (second level) (see Figure 9). By using the Move4D scanning system, the temporal change of the airgaps is shown as a function of the movement. In order to merge the scan data, identical points in the scan data must be found and translated. Since it is not possible to execute the exact same pose twice, the scan data cannot be fitted into each other without deviation. The scan data are compared once in the form of polygonal areas and in the form of cross-sectional areas at defined heights as shown in Figure 1(Fig. 1). The comparison of the two clothing levels, especially in the leg region, shows that the scanning method of the Move4D system is suitable for the analysis of airgaps.

## Initial Testing Results

### Improving the visibility of reflective areas

3D and 4D scanners are sensitive to the surface, which they scan - they need reflected light in order to detect the positions on the surface. The type of the yarn interlacement of the textile structures will influence the surface geometry and the reflection of the light; the surface treatment (calendering) and the use of reflective coatings and foils can significantly disturb the work of the scanning modules. Both factors (structure with smooth surface in twill pattern and reflective elements) are present in firefighters' clothing and led to problems during the scanning.

**Figure 2:**
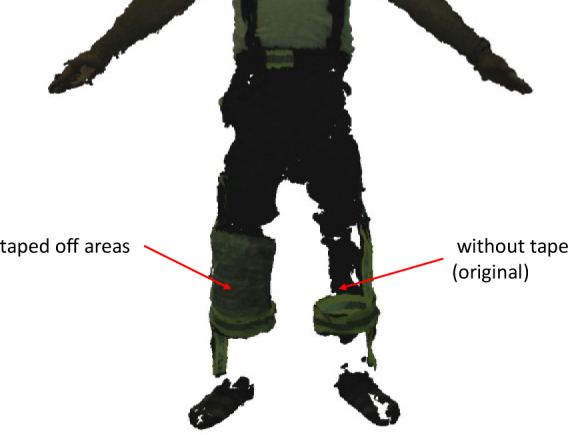
Point cloud after the 4D scan with taped areas and with the original material of the firefighters' trousers.

Figure 2(Fig. 2) shows a point cloud in firefighters' clothing obtained by the Move4D scanner in an A-pose. The A-pose is a standard reference pose commonly used in 3D scanning of human models. During the first scans, there were significant gaps in the scans, especially on the reflecting areas. For this reason, these areas were masked off to reduce gaps. The right side of the image showed very large gaps at the knee level. Here, the 4D scanner could not capture any points because of the material. As a countermeasure, the material areas were covered with tape, which significantly reduced the gaps. Points were present on the left side of the knee. These will require further processing later.

**Figure 3:**
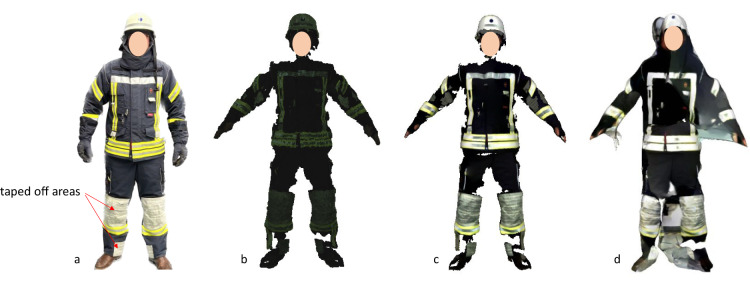
a) Photographed firefighter clothing with areas partially taped off; b) Scanned A-pose to capture point cloud using 4D scanner; c) Calculated mesh from point cloud; d) homologous mesh

The non-recognized material prevailed in the areas of the knees and elbows, as well as in the area of the shoes. According to consultation with the clothing manufacturer, the problematic material is a reinforcement with a special silicone coating. We were not given more precise specifications. For this reason, these areas were masked off. Despite the covering, the scanner could not capture the entire clothing in the form of a point cloud. As can be seen in Figure 3b(Fig. 3), larger gaps were always visible. A sufficient and good quality point cloud is a prerequisite for the creation of a 3D model. A point cloud is a collection of points in three-dimensional space representing the surface of a scanned object or environment. Each point in the cloud contains information about its position in space and possibly its color or texture. If there are not enough points, a closed mesh of the model cannot be created, where a mesh is a collection of shapes that represent the surface of the entire object. These surfaces are created by connecting points in the cloud to form a three-dimensional mesh of triangles, squares or other geometric shapes. Figure 3c(Fig. 3) shows the calculated mesh based on the captured points. Again, large gaps can be seen which did not facilitate to adequately parameterize the firefighters' clothing. The attempt to automatically close the gaps with the software provided with the 4D scanner was unsuccessful, leading to significant distortions and an unrealistic output. Figure 3d(Fig. 3) shows the calculation of the algorithms that attempted to calculate a homologous mesh. A homologous mesh is a 3D model that consists of two or more similar geometric shapes or objects that have the same topology and number of vertices, edges and faces. In this case, a human model was taken as the basis.

### Motion analysis

**Figure 4:**
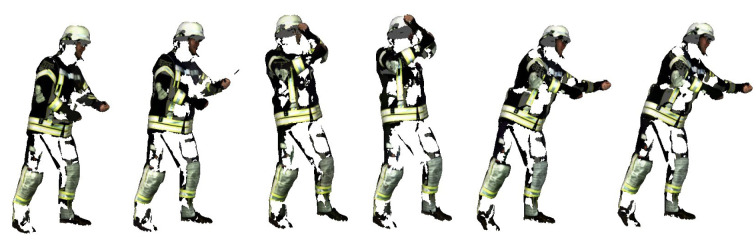
4D-Scan recording a movement using a Halligan tool

In addition, various movements such as walking, running, lifting or rescuing people were recorded in real time with the 4D scanner for further evaluation in further studies. All movements were performed with the same clothing and by the same participant. The method presented in this study was initially performed for only one movement in order to demonstrate the method. Figure 4(Fig. 4) shows an exemplary movement in which the participant used a Halligan tool. This is a special tool used by firefighters to open or break through doors, windows and other barriers in rescue and entry scenarios. It consists of a curved rod with a flat tip at one end and a curved claw at the other. A movement and use of the Halligan tool was applied simulating a situation when uncovering a wall. The Halligan was moved above the head so that it penetrated a fictitious wall and then it was pulled to tear off, for example, a wooden panel exposing possible fire sources. The movement of using the Halligan tool was recorded three times through the 4D scanner. For further analysis, only the one with the fewest errors (missing areas, distortions, overlays, and texture errors) was selected. The same locations of gaps in the point cloud occurred in the scanned movements. The other movements (walking, running, lifting and saving a person) will be analyzed in further studies. 

After processing the scan of the movement, larger gaps were visible, which made it difficult to evaluate the clothing geometry during the movement. There were too few points at the corresponding locations for accurate measurements assessing the change in position of the clothing. The probable reason for this was that the clothing absorbed or reflected the light of the cameras and sensors.

### Cloud comparison

Each movement is composed of individual images and consists of individual point clouds. The individual point clouds can be displayed in different colors and provide detailed information about the position of the protective clothing on the body in different poses. The observation of the frame images of a motion on point cloud level was not simple, because behind the points of the front size also those of the opposite sides were visible. For better observation, the size of the points in the 3D visualization had to be adjusted to create a cloud without gaps between the points. Checking the Gromov-Hausdorff distances between the different images helped to visualize the movement of the cloud of points. 

**Figure 5:**
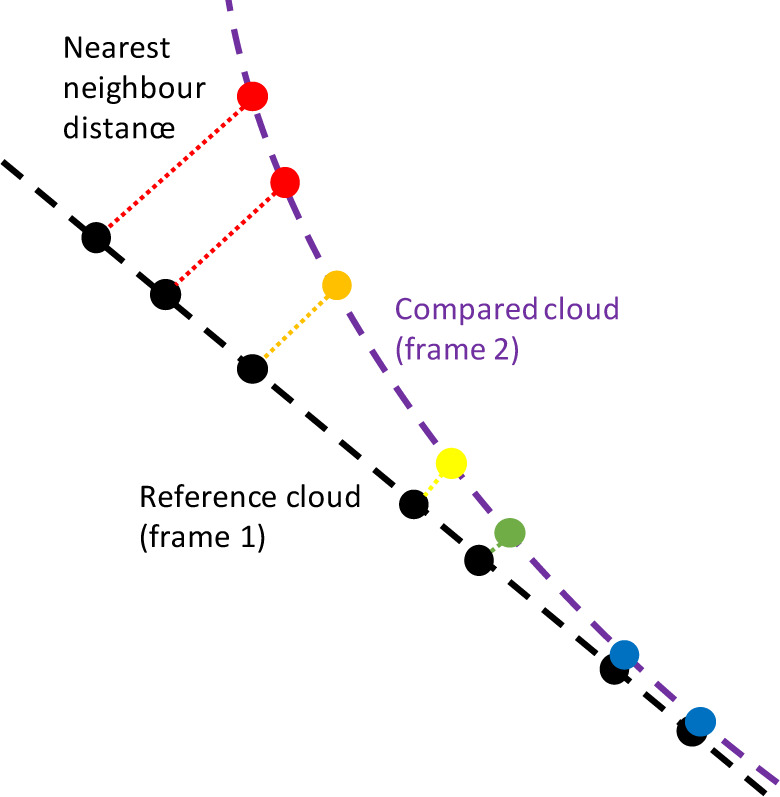
Schematic illustration of the point-to-point distance determination according to Gromov-Hausdorff

The Gromov-Hausdorff distance is a measure that quantifies the distance between two metric spaces. Schematic representation of the point-to-point distance determination is shown in Figure 5(Fig. 5). Using the distances between the individual cloud points, the areas that did not move (blue color) and the areas where a larger displacement was detected (from green to red) could be visualized.

**Figure 6:**
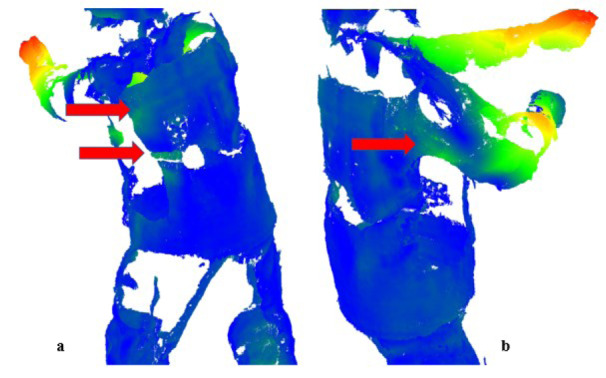
Point cloud (4D scan) of a person wearing firefighter protective clothing - computed Gromov-Hausdorff distance between two clouds (100 milliseconds of motion) and marked yellow/red areas indicating larger distances

However, the present case did not really provide useful information about the areas where the protective clothing affected the body in an uncomfortable way. The distances between the individual images can be used to visualize the areas that do not move (blue colour, Figure 6a and b(Fig. 6)) and the areas where a larger displacement is detected (green - red, Figure 6(Fig. 6)). Normally, the protective clothing is assumed to move with the body, but a scan without clothing and a scan with clothing are required to detect this relative movement. Unfortunately, it is not possible for a human to perform a movement twice in an absolutely identical manner. Using the 4D scan to create an accurately realistic movement avatar of the usual movements of humans without protective clothing and then using these avatars with clothing simulation software would probably be the best method for analyzing the relative movement between humans and protective clothing.

**Figure 7:**
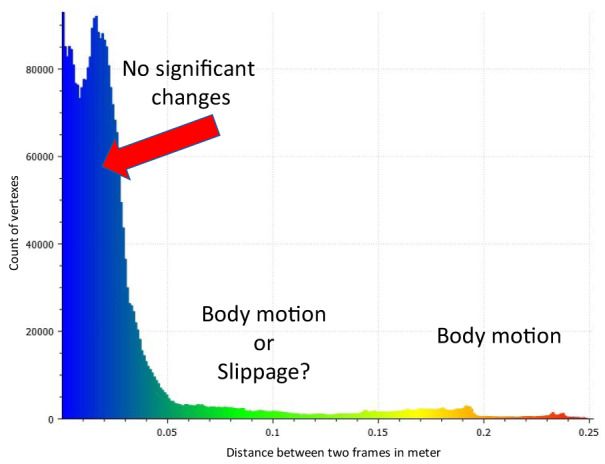
Distribution of the distances between points in two frames of a motion. X axis - distance in meter, Y axis - number of vertexes at the corresponding distance to the closed area from the second mesh

Figure 7(Fig. 7) shows the number of faces with distance referring to the actual distance from point to point, as shown in Figure 5(Fig. 5). The absolute distance does not take into account detours or intermediate stops but measures the shortest line connecting the points. In this regard, it can be noted that it was difficult to determine exactly what is happening based solely on the distance between two frames and the meshes. The large distance (red) of more than 10 cm was connected with the human body motion, indicated by the arms positions in Figure 6(Fig. 6). The major part of differences between the meshes were below 3 cm (marked blue).

Checking the areas with “uncommon” color, areas could be identified where the clothing movement differed considerably from the environment, which could indicate potential problems with the freedom of the body mobility. 

**Figure 8:**
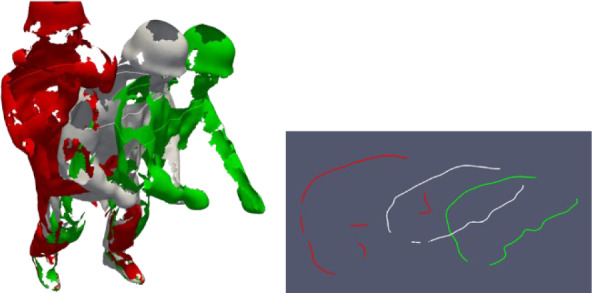
a) 3 Frames of motion with the Halligan tool; b) Cross section profiles of each frame

The limited visibility of the clothing produced scans with many gaps, impeding the evaluation of distances or cross sections. Figure 8a(Fig. 8) shows 3 frames obtained during the motion and on the right (Figure 8b(Fig. 8)) the cross-section profiles at that level. The non-visible areas in the cross section hampered the computation of circumferences or the definition of areas for tracing curves in the 3D data sets.

### Alternative methods

As an alternative approach to obtain 3D geometry data for posture evaluation, the Artec Leo 3D hand scanner (Artec 3D, Luxembourg) was tested. A participant was again scanned in a static A-pose. The handheld scanner, which is based on structured light technology and has a wide field of view, captured 3D data by projecting structured light onto the subject to generate a 3D model. The pictures from the hand scanner looked less distorted compared to the result from the 4D scanner, however, there were considerable differences between both techniques in the scanning performance data (Table 1(Tab. 1)). The handheld scanner generated one 3D image based on 18 frames per second and an experienced scanning person took 6 minutes to complete the procedure. This is equivalent to accumulating information from 8640 3D frames into one image in 3,7 million of vertices. If the human was not moving, this data would be precise, but natural muscle activity and breathing caused errors of more than 1,5 cm in the position (Kyosev and Siegmund, 2019[15]). On the other hand, the scanning time at Move4D is a small part of the second, eliminating all these disturbing motion effects. The processed mesh with triangles has 77 976 faces, which is small compared to the 100 times larger size of the mesh from Leo 3D scanner. Figure 4(Fig. 4) and Figure 6(Fig. 6) indicated that the mesh from Move4D provided sufficient details and a satisfactory mesh size, if the larger areas of reflective elements were visible. 

*S*_[m_^2^_]_ = 0,0235 * *L*^0,42246^ • *M*^0,51456^ (Eq. 1)

**Figure 9:**
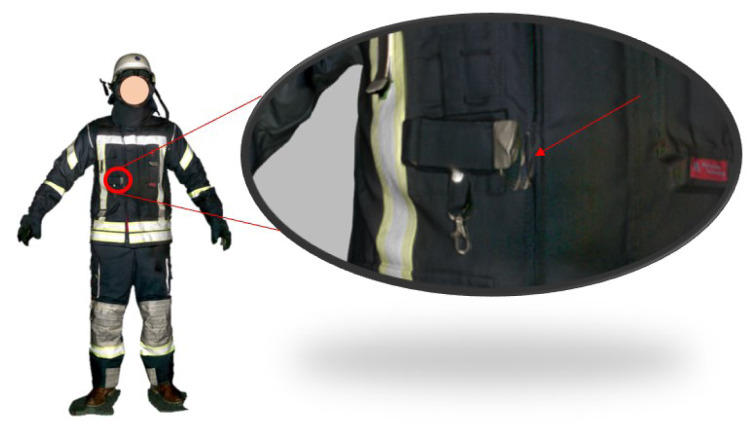
Scanning result with the Leo hand scanner and a marked shifted area

The nude body surface area *S* of the scanned human amounted to 2,08 m^2 ^according to Eq. 1 (Gray et al. 2015[10]) calculated from the body height *L* (in cm) and body mass *M* (in kg). The comparison with the 1,5 m^2 ^ resulting from the Move4D scan showed that approximately 0.5 m^2 ^of the surface was not visible, due to gaps. The precise scan data of Leo 3D yielded 3,395 m^2 ^surface area, computed in MeshLab, representing an overestimation by 1,4 m^2 ^or 67 % (Table 1(Tab. 1)). Figure 9(Fig. 9) shows that the small clothing details like pocket lids, belts etc were saved accurately by the handheld 3D scanner, which increased the measured surface. The non-visible areas were closed using the algorithms for closing holes in the MeshLab software, based on the algorithm (Liepa, 2003[16]).

Although the accuracy of the 3D Leo scanner could be useful for static digitalization of clothing, it was not considered suitable for the analysis of the mobility. For this purpose, the Move4D data will be used, because it provides the information in the time domain with higher accuracy and more efficient storage size.

## Conclusions and Outlook

### Conclusion

The preliminary tests within this study showed that firefighters' clothing material and its reflective properties cause problems in detection by 4D scanners. This could lead to gaps in the point cloud and caused difficulties in the examination of the clothing during movement. Although an alternative technique using a handheld 3D scanner provided better static results, these scanners are not suitable for examining movement. The imaging in the 4D scanner of the critical regions (in this case the elbows, knees) need higher accuracy. To achieve this, the algorithms for detection would have to be improved, or the non-detectable material would need replacement, although this would change the overall property of the protective clothing and would not represent the true clothing behavior. There is a need for further studies using the 4D scanner for the determination of material or coating properties hampering the detection during scanning, in order to improve the scanning method in this regard.

### Outlook

Future studies should aim to further improve the recording and analysis of firefighters' clothing. This could include the study of clothing materials that are less reflective or absorbent. In addition, techniques could be developed to analyze the relative movement between humans and protective clothing, such as creating accurately realistic movement avatars without protective clothing and using clothing simulation software. In addition, further work should aim to develop algorithms for automatic analysis of clothing deformation so that the full information from high-speed body scanning can be objectively analyzed and used to develop improved protective clothing. For the objective evaluation of protective clothing, a subjective evaluation (in the form of survey) using human participants will also be included in our study in a next step, together with the 4D scan, in order to be able to improve the assessment of the wearing comfort.

## Acknowledgements

This investigation was partially performed within the frame of IGF research project 21622 BR of the Forschungsvereinigung Forschungskuratorium Textil e. V., funded through the AiF within the program for supporting the „Industriellen Gemeinschaftsforschung (IGF)“ from funds of the Federal Ministry for Economic Affairs and Climate Action on the basis of a decision by the German Bundestag.

## Figures and Tables

**Table 1:**
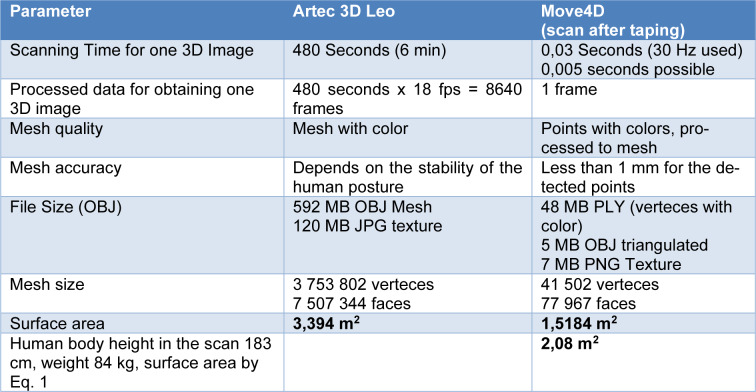
Comparison of the scanned data sets with Artec Leo and Move4D
